# The impact of melt versus mechanical wear on the formation of pseudotachylyte veins in accretionary complexes

**DOI:** 10.1038/s41598-022-05379-5

**Published:** 2022-01-27

**Authors:** B. Moris-Muttoni, H. Raimbourg, R. Augier, R. Champallier, E. Le Trong

**Affiliations:** Institut des Sciences de la Terre d’Orléans, Univ. Orléans, CNRS, BRGM, ISTO, UMR 7327, 45071 Orléans, France

**Keywords:** Geodynamics, Structural geology, Petrology, Tectonics

## Abstract

Whether seismic rupture propagates over large distances to generate mega-earthquakes or is rapidly aborted mainly depends on the slip processes within the fault core, including particularly frictional melting or intense grain-size reduction and amorphization. The record of seismic slip in exhumed fault zones consists in many instances in Black Faults Rocks, dark and glass-like-filled aphanitic veins that have been interpreted as resulting from the quenching of frictional melts, i.e. pseudotachylytes. Such interpretation has nevertheless been questioned as similar macro to nano-microstructures have been observed either on intensely comminuted natural fault rocks or on slow creep experiments conducted on crustal rocks, where melting is absent. Here, we report a new dataset of Raman Spectroscopy of Carbonaceous Material analyses, aimed at discriminating the slip weakening processes operating in the fault core during slip. Using high spatial resolution profiles on natural Black Fault Rocks from exhumed accretionary complexes and an experimentally calibrated modelling of Raman intensity ratio evolution with temperature, we assessed different scenarios of temperature evolution during fault slip. None of them is able to account for the distribution of Raman signal, so that in the three studied Black Fault Rocks interpreted so far as natural pseudotachylytes, Raman Spectroscopy of Carbonaceous Material rather reflects the effect of intense and localized strain during fault slip. Furthermore, the absence of thermal imprint on Raman signal puts upper bounds on the temperature reached within the fault zone. If one cannot rule out the occurrence of high and short-lived temperature increase due to friction, the latter was not high enough as to melt the large quartz fraction of the fault zone rocks.

## Introduction

Pseudotachylytes have been described for the first time at the beginning of the twentieth century as veins filled with black and aphanitic to glassy material^1^. They are considered as resulting from frictional melting produced by landslide, meteor impact, or fault rupture^2–4^, most often in the shallow and brittle domain of the lithosphere, but in some instances at important depths until to the eclogite facies conditions^5–7^. Many pseudotachylyte veins are described within fault systems in basement crustal rocks and are associated with different types of brittely deformed rocks such as cataclasite or fluidized gouge. Pseudotachylyte veins present typical features such as injection structures, ultra-fine matrix or microlites growing at the expense of the melt^5^. More recently, Black Fault Rocks have been described in sedimentary accretionary prisms^8–14^ with macro- and micro-structures similar to the pseudotachylytes reported in basement crustal rocks^5^. These Black Fault Rocks are described as fault core filling material in zones of intense deformation of the host-rock at low to very low temperature conditions ranging from 160 to 320 °C.

### Ambiguity of the microstructures

Whether Black Fault Rocks originate from friction melting or mechanical wear and drastic grain-size reduction is a long-standing issue, due the ambiguity of some of the microstructures associated with pseudotachylytes^5^. Indeed, micro-structural features, such as fluidization and flow textures, ultra-fine matrix and aphanitic textures, or corroded clasts were considered as a result of ultra-comminution^15–20^ or intense fluid-rock interaction within the fault core^21^, without melting. This issue is even more acute in the Black Fault Rocks hosted in low-grade metasediments, which contain large amounts of mineral-bound water: upon slip and temperature increase, thermal pressurization of released water might reduce friction and prevent from further temperature increase and ultimately from melting^22–24^.

### Insights from RSCM on fault slip processes

Raman Spectroscopy of Carbonaceous Material is a powerful geothermometer widely used in metamorphic petrology to constrain peak-temperature conditions using the strong dependency of carbonaceous material crystallinity with metamorphic temperatures^26^. This tool has proven very reliable for both regional and contact metamorphism^25–27^. More recently, Raman Spectroscopy has been also used in order to evidence frictional heating during seismic events on natural Black Fault Rocks from accretionary complexes^28–31^ and on experimentally deformed samples^28,32^. However, crystallinity of carbonaceous material in intensely deformed rocks may be also influenced by parameters other than temperature. In particular, shear has been shown to be responsible for a large increase in crystallinity of carbonaceous material^33,34^ even if in some other examples, the relationship between strain and Raman spectrum evolution is more ambiguous ^28,30,31,35^. Therefore, in Raman Spectroscopy studies on both naturally and experimentally damaged rocks, it is difficult to disentangle thermal and mechanical effects.

A way out of this conundrum relies on the fundamental difference between temperature and deformation as the strain field might be discontinuous, while the temperature field, because of diffusion, is not. Based on this straightforward idea, we developed a new high-resolution spatialized approach to Raman Spectroscopy (*i.e.* spot size < 2 µm), to unravel how the carbonaceous material crystallinity is distributed in space within rock samples. We applied this approach to three Black Fault Rocks described as pseudotachylytes in two exhumed accretionary complexes, two in the Shimanto Belt^8,12^ in Japan and one in the Kodiak Accretionary Complex^13,14^ in Alaska, in order to discriminate the respective effect of temperature increase and strain, and eventually to decipher the mechanical processes at the origin of the Black Fault Rocks.

## Results

### Structures

**Figure 1:**
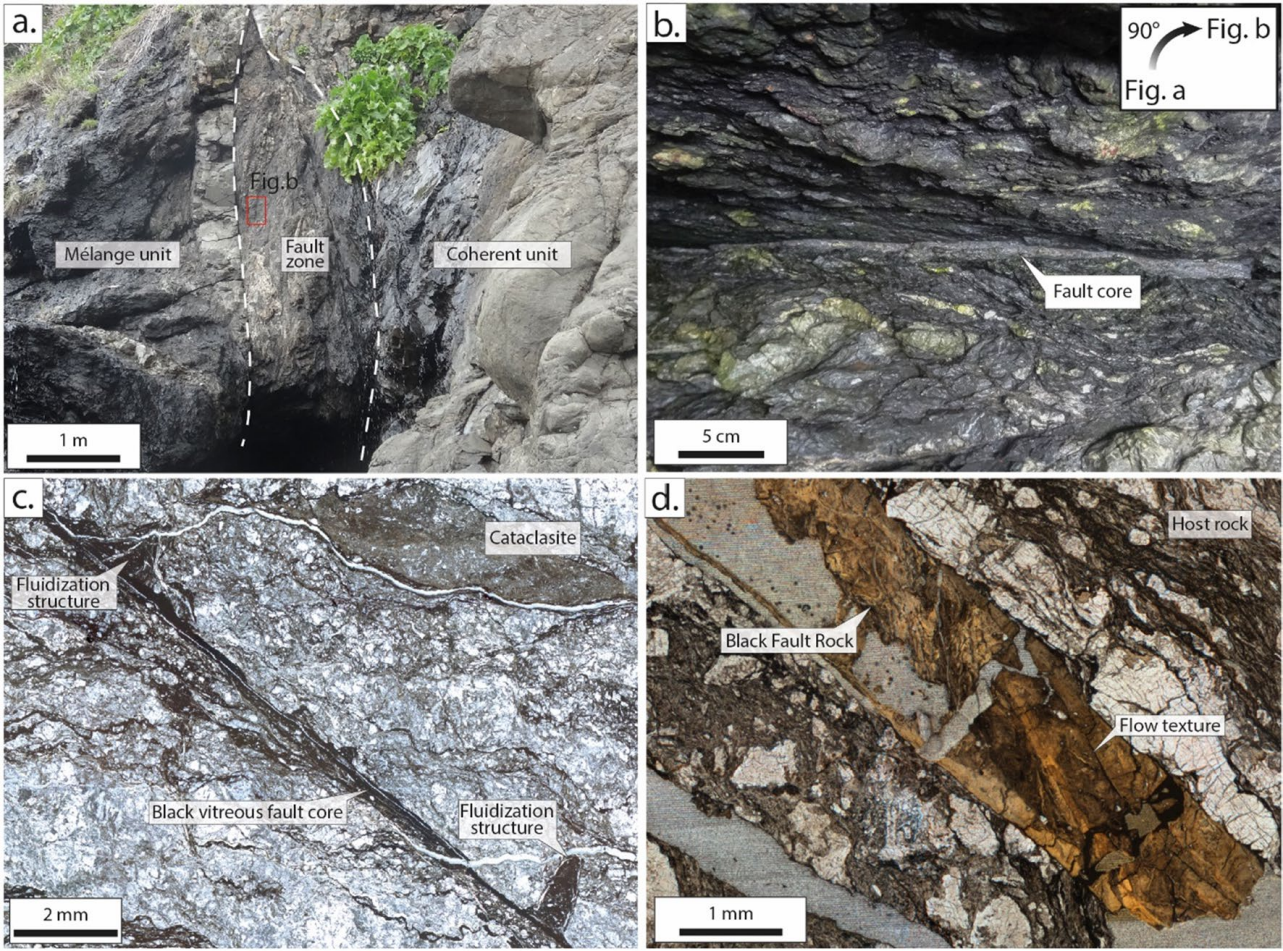
Structures and micro- to nano-structures of the Black Fault Rocks observed in fossil accretionary complexes. (**a**) Meter-thick fault zone between the Mugi mélange unit and a coherent unit in the Shimanto Belt (**b**) Close-up view of the fault zone with the fault core (**c**) Fluidization structures (embayment and injection vein) and Black Fault core in the Okitsu Black Fault Rock (**d**) Flow textures inside the Nobeoka Black Fault Rock Locations: (**a**,**b**) Mugi mélange (**c**) Okitsu mélange (**d**) Nobeoka Tectonic Line drilling.

**Figure 2:**
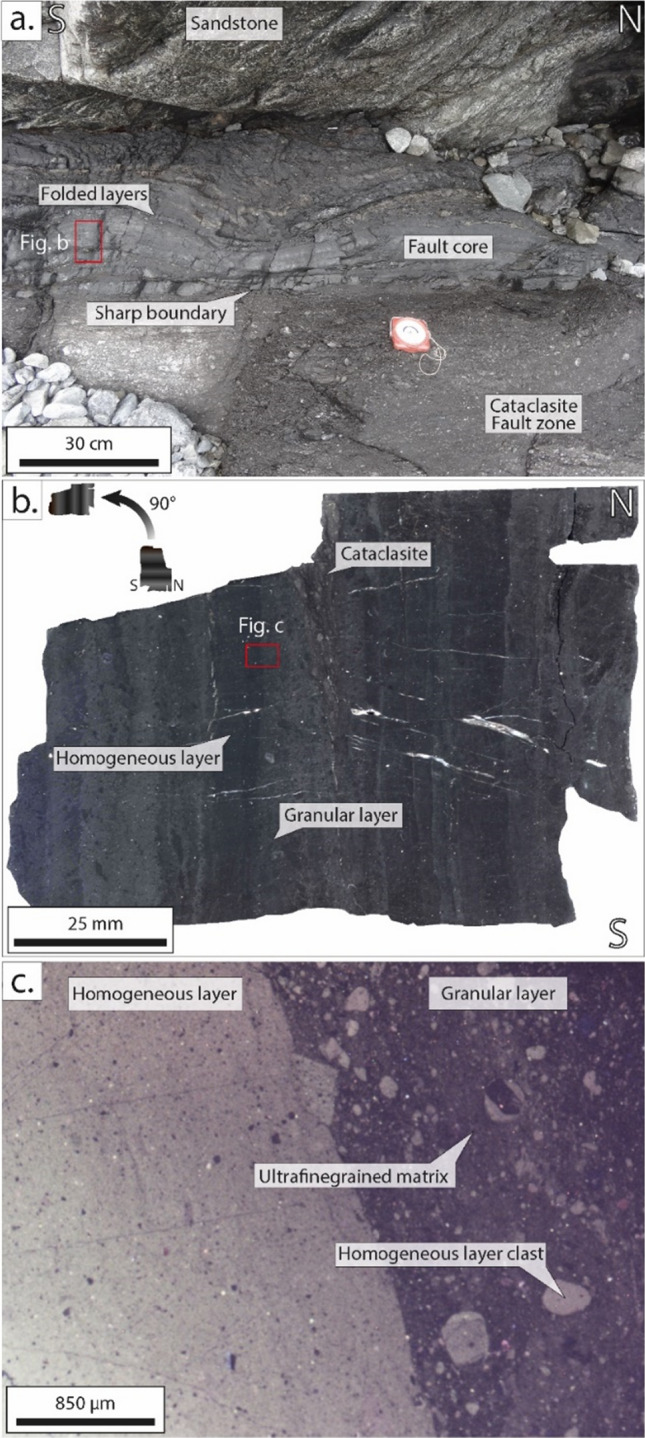
Structures and microstructures of a Black Fault Rock from Kodiak Island highlighting the multi-layering (Alaska, USA). (**a**) Outcrop in the Kodiak Accretionary Complex showing the aphiric fault core sharply cutting the host rock and folded in a later stage (**b**) Internal structure of Kodiak Black Fault Rocks showing alternations of homogeneous and granular layers (**c**) Cathodoluminescence image of Kodiak Black Fault Rock, highlighting the difference between the granular and the homogeneous layers and showing clasts of the homogeneous layer embedded in an ultrafine-grained matrix.

The two exhumed accretionary complexes investigated here were both formed by stacking of tectonic units composed either of coherent turbidites or tectonic mélange units, both mostly composed of shales and bounded by thrust faults^36,37^. Some of these fault zones, contain Black Fault Rocks bearing characteristic features of pseudotachylytes^8,12–14^. Black Fault Rocks are black and aphanitic thin veins, which cut sharply across, with straight boundaries, the host-rock that often contains itself brittle microstructures (Fig. 1). The two Black Fault Rocks from the Shimanto Belt are millimeter-thick or less but in Kodiak, the Black Fault Rock described by Rowe et al.^13^ reaches up to 30 cm in thickness. In addition, in contact with meter-sized sandstone lenses, some Black Fault Rocks show structures interpreted as injection structures of a fluidized material perpendicular to the fault core^8,14^ (Fig. 4a–c). The filling material is also black and has an aphanitic texture (Fig. 2).

### Microstructures

**Figure 3:**
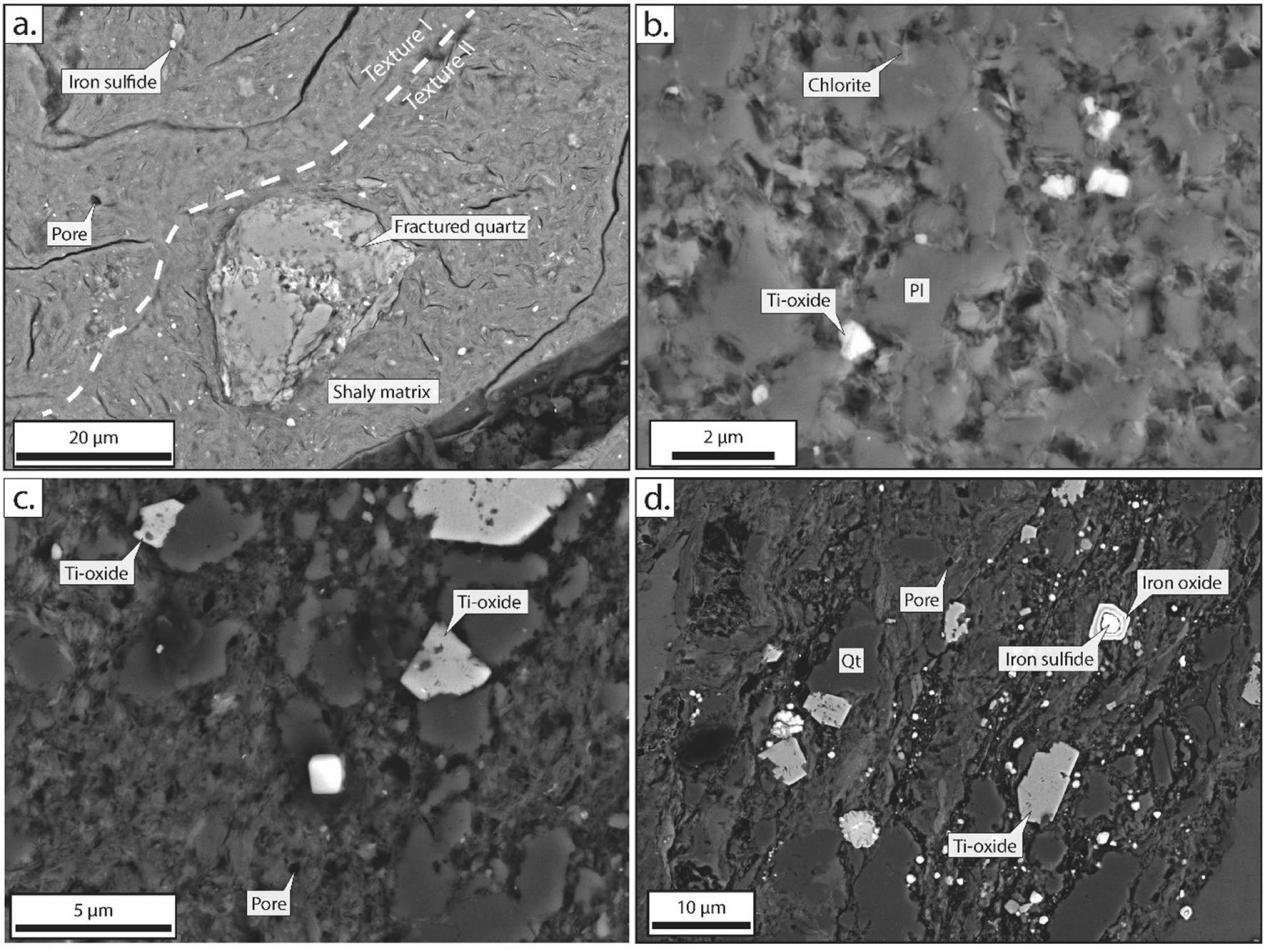
SEM illustrations of the microstructures observed in the Black Fault Rocks and its host-rock. (**a**) Fractured quartz in a shaly matrix containing scattered iron sulfides and pores and showing two textures defining layering ; (**b**) (Sub)euhedral titanium oxides in a Black Fault Rock composed of plagioclase clasts and a chlorite matrix (**c**) Shaly matrix of the Black Fault Rock containing euhedral titanium oxides and pores (**d**) Host-rock shaly matrix containing quartz clasts and iron sulfides replaced by titanium oxides on the rim Location (**a**), Nobeoka Black Fault Rock; (**b**) Kodiak Black Fault Rock; (**c**,**d**) Okitsu Black Fault Rock. *Qt* quartz, *Pl* plagioclase.

The Black Fault Rock material is characterized by sub-rounded to rounded quartz clasts, feldspars (mostly albite), and sometimes calcite^8^, less than 100 µm in size, embedded in a ultra-fine black matrix. Clasts, sometimes, show embayment, corroded and fractured structures^12^. Clasts are wrapped in a matrix (Fig. 3a,b). Under SEM, the matrix seems composed of an ultra fine-grained material composed mostly of phyllosilicates, with the notable occurrence of pores^10,13^, micrometer scale scattered euhedral iron sulfides or titanium oxides^8,12,14^ and scattered angular clasts (Fig. 3c). For example, the Kodiak Black Fault Rock is composed of 1 to 2 µm euhedral zoned plagioclases, in a matrix composed of acicular chlorite^14^ (Fig. 3b). Iron sulfides and titanium oxides present euhedral to sub-euhedral shapes and sizes ranging from ca. 0.5 to 1 µm (Fig. 3b,c). In rare cases, framboïdal iron sulfides are observed in the BFR layer (Supplementary Fig. 4). Titanium oxides are mostly observed in the homogeneous layers, whereas iron sulfides are more abundant in the coarse-grained layers. In the Nobeoka fault zone, Black Fault Rock observed in optical microscopy and SEM show flow textures, where the layering is defined by the distribution of sulfide grains^12^ and the quantity and the shape of pores (Figs. 1 and 3a), while the ultrafinegrained matrix and clasts remain homogeneous. This microstructural evolution is also observed in Okitsu and Kodiak Black Fault Rocks. However, on the extreme rim of the Nobeoka Black Fault Rocks, a smaller grain size of the clasts in the ultrafinegrained matrix is observed.

The host-rock of the Black Fault Rocks (Fig. 3d), is also composed of quartz and albite clasts embedded in a matrix composed of phyllosilicates with scattered iron sulfides and titanium oxides, the latter with a much lower abundance than in the Black Fault Rock. The grain size of quartz and albite clasts is larger than in the Black Fault Rock. Clasts embedded in the matrix show angular shapes and internal fractures. Corroded shapes on quartz and albite are often observed (Supplementary Fig. 4). Titanium oxides have euhedral shapes, while the iron sulfides have either euhedral or framboïdal shapes. Iron sulfides show a clear metamorphic coronitic reaction including the production of iron oxides (Fig. 3d).

### Polystaged vs single-staged

Moreover, two types of microstructures have been observed for these Black Fault Rocks. Samples from the Shimanto Belt are composed of a single homogeneous layer. In contrast, the thicker Black Fault Rock from Kodiak has a multi-layered structure, with “homogeneous” or “granular” layers, visible in both proportion and size of the clasts embedded in the vitreous matrix (Fig. 2). Homogeneous layers are composed principally of an ultrafinegrained matrix, bright in cathodoluminescence imaging, embedding scattered, micron-size, particles. Granular layers are composed of an ultrafinegrained matrix, dark in cathodoluminescence imaging, embedding a large proportion of clasts of various sizes from the homogeneous layer (Fig. 2c). These features support the hypothesis of a polystaged formation process for the Kodiak black Fault Rock.

### RSCM profiles

**Figure 4:**
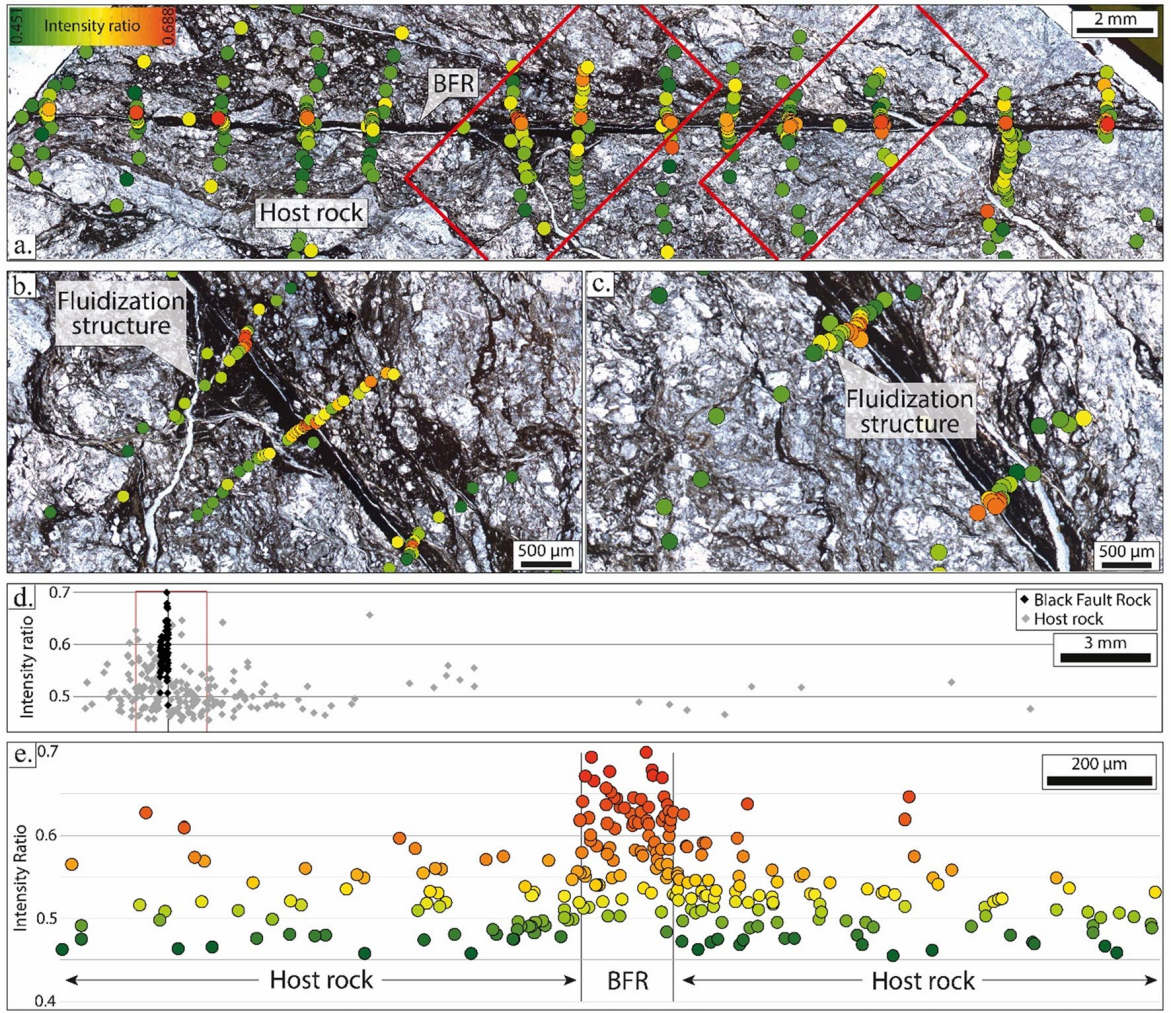
Raman spectroscopy results obtained for the Okitsu Black Fault Rock and its host rock. (**a**) Intensity ratio evolution along high-resolution cross-sections of the Black Fault Rock; (**b**,**c**) Close-up views of the fault core and the fluidization structure. (**d**) Integrated cross-section showing RSCM intensity ratio evolution. (**e**) Close-up view (red square in (d)) which shows the sharp evolution of the RSCM intensity ratio near the fault core. *RSCM* Raman Spectroscopy of Carbonaceous Material.

**Figure 5:**
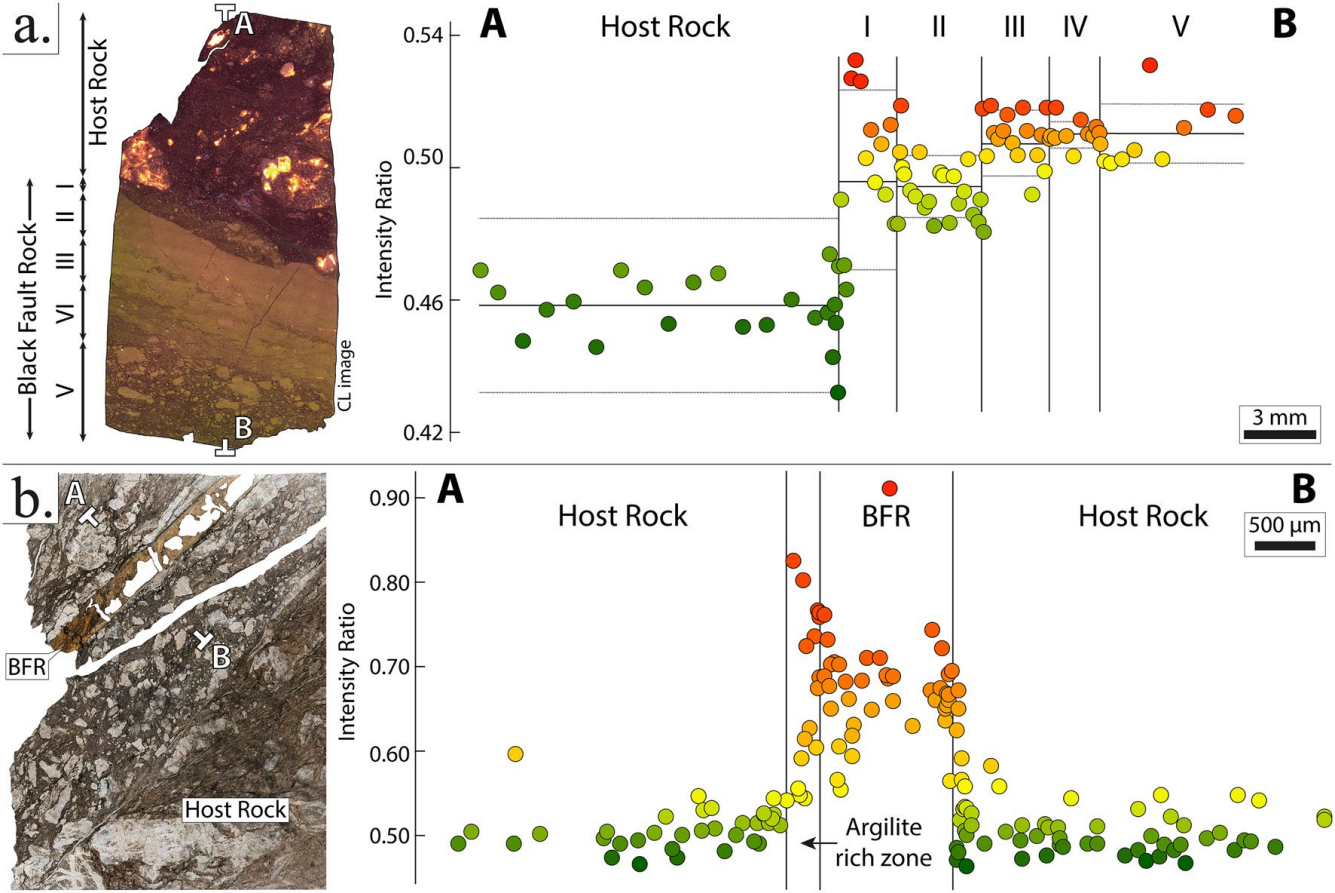
Raman spectroscopy intensity ratio and microstructures of the Kodiak (**a**) and Nobeoka Black Fault Rocks (**b**) and their host rocks. Note that in (**b**), the Black Fault Rock is composed of several layers labelled I to V.

In this study, the Raman Spectroscopy intensity ratio of carbonaceous material (i.e., R1 in Beyssac et al.^26^) and area ratio (i.e., RA1 in Lahfid et al.^38^) were measured over high-resolution cross-sections through the Black Fault Rocks and the adjacent bounding host-rocks. The intensity ratio corresponds on the intensity on the raw spectrum of the defect band (i.e. centered at 1350 cm^−1^) divided by the intensity of the graphite band (centered at 1600 cm^−1^). Intensity ratio evolutions, along each cross-section (straight lines or synthetic cross-sections compiling the projection of distinct segments), show a significant increase, revealing a higher crystallinity inside the veins (Figs. 4 and 5; Supplementary Figs. 1 and 2). The median intensity ratio in the host-rock and the Black fault Rock is 0.500 and 0.600 (Okitsu), and 0.500 and 0.682 (Nobeoka), respectively. In Kodiak, the median intensity ratio in the host-rock is 0.454, while it reaches 0.497, 0.490, 0.507, 0.508 and 0.505 in the five layers forming the Black Fault Rock, labelled I to V, respectively. In the Kodiak Black Fault Rock, the highest median intensity ratio values were not analyzed on the homogeneous layer II but on the granular layers with clasts-and-matrix structures layers III to V. Most importantly, intensity ratio profiles show a discontinuity at the boundary between the Black Fault Rocks and the host-rock jumping from 0.530 to 0.620 in Okitsu, from 0.520 to 0.680 in Nobeoka and from 0.490 to 0.530 in Kodiak (using a local median on zones a few micrometers-wide on each side of the boundaries). Within the Black Fault Rock, the highest values are mostly observed near the boundary with the host-rock, within a zone a few micrometers wide (Figs. 4 and 5) (Supplementary Figs. 1 and 2). Moreover, intensity ratio values measured inside the Black Fault Rocks show an important scatter marked by a higher standard deviation, from 0.010, 0.004 and 0.009 in the host-rock to 0.063, 0.052 and 0.017 in the Black Fault Rocks of Nobeoka, Okitsu and Kodiak respectively. In addition, extremely high intensity ratios are present inside the Black Fault Rocks, up to 0.92 for the Nobeoka Black Fault Rock, 0.72 in Okitsu and 0.55 in Kodiak. Finally, fluidization structures described as injection veins show similar intensity ratios and standard deviation values 0.529 ± 0.019, as the ones in the host-rock, hence a lower intensity ratio than the Black Fault rocks (Fig. 4).

### Thermal modelling of RSCM evolution

#### Model description

In order to interpret the Raman Spectroscopy ratio profiles across Black Fault Rocks, we developed a model to evaluate the contributions of the effects of heat production and temperature increase during fault slip on carbonaceous material crystallinity. This model combines (1) heat diffusion and (2) kinetics of carbonaceous material evolution during carbonization and early graphitization processes.

Kinetics of Raman Spectroscopy of Carbonaceous Material signal evolution as a function of temperature was calibrated on the basis of thermal maturation experiments, following Nakamura et al. procedure^39^ (Experimental procedure are developed in Supplementary Note 2).

Heat diffusion modelling is based on the heat equation applied to a 1D system, composed of an infinite matrix embedding a molten layer of thickness *h*, (= the current thickness of the Black Fault Rock). We considered three idealized end-member cases to model the evolution of the system before and after melting. Case (1) corresponds to a fault zone of thickness h, where heat is dissipated by mechanical work, up to melt the central portion of the fault. Heat production is described as $\dot{E}= \tau .\left(\frac{u}{h}\right)$, with $\tau$ (shear stress) and $u$ (slip velocity) inferred from geodetic and seismological models^40^. Cases (2) and (3) describe the temperature field in a molten layer of thickness h, without/with viscous shear heating, respectively. Heat production is modelled as $\dot{E}=\mu {\left(\frac{u}{h}\right)}^{2}$, where $\mu$ is the melt viscosity.

Slip parameters are subject to large uncertainties, even if mechanical and seismological data provide some constraints on the orders of magnitude. Different slip models, in terms of velocity $u$ and total duration, were considered, in order to fit the intensity ratio measured in natural rocks.

The geometry and boundary conditions of the model were adjusted to the natural case study of the Nobeoka Black Fault Rock. The thickness of the molten layer of the modelling is fixed to 1 mm. The initial temperature and intensity ratio field are very uniform and equal to 200 °C and 0.50, respectively. The temperature of the molten layer is fixed at 1400 °C, based on the interpreted melting temperatures for Black Fault Rocks in accretionary complexes and experimental reproduction of molten origin BFR in the literature^11,14,20,41^. Complementary information are given in the Supplementary note 1 and 2.

### Modelling results

The temperature evolution before melting (Case (1)) is extremely short, as the frictional melting is reached after only few hundredths of second. For this short heating time, the evolution of the intensity ratio of Raman spectra, around 0.003, appear not significant (Supplementary Fig. 3).

After melting at 1400 °C, if viscous shear heating is negligible (Case (2)), inside the molten layer the temperature drops down very quickly (in few milliseconds) so that at the center of the layer, the temperature is reduced to 800 °C after less than 1 s. In the host-rock, heat diffusion leads to an increase of temperature, locally up to 800 °C, but for a very short time. In this scenario, the intensity ratio reaches 0.62 in the center of the molten layer and decreases rapidly laterally, down to unchanged values of 0.50 at the boundary with the host-rock.

**Figure 6:**
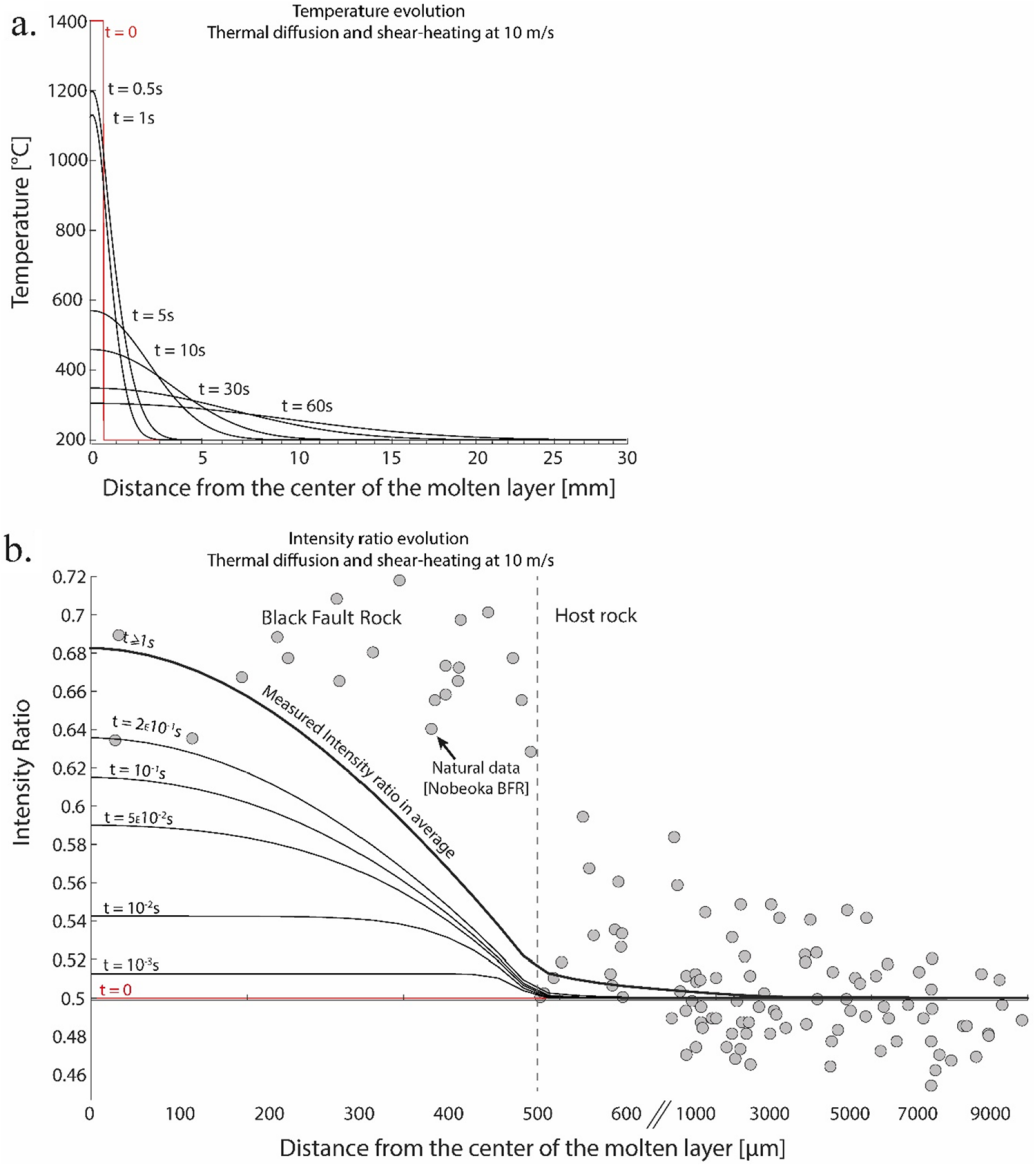
Thermal and kinetic modellings obtained for a 1 mm-thick molten layer where heat is transferred by diffusion to the host rock and is produced in the molten layer by viscous shear heating. (**a**) Temperature and (**b**) Intensity ratio evolution at different times (curves). Also presented, intensity ratio of the Nobeoka Black Fault Rock. Slip parameters (slip rate and total slip) were adjusted so that the modelled intensity ratio reaches the natural values at the center of the molten layer.

The significant intensity ratio increase in the molten layer requires therefore incorporating the production of heat by viscous shear as the fault is slipping (Case (3)). We adjusted the slip velocity to 10 m/s and the duration to 1 s so that the modelled intensity ratios in the center of the molten layer are equal to the natural ones measured in Nobeoka Black Fault Rock (ca. 0.68; Fig. 6). In spite of significant heat production by viscous shear, the diffusion of heat away from the molten layer into the cold host-rock is extremely efficient, so that sharp temperature and intensity gradients develop exclusively within the molten layer (Fig. 6a). Therefore, in the outer domains of the molten layer, the intensity ratio remains the same as host-rock values throughout slip (Fig. 6b).

## Discussion/conclusions

### Shape of modelled vs. natural RSCM ratio profiles

**Figure 7:**
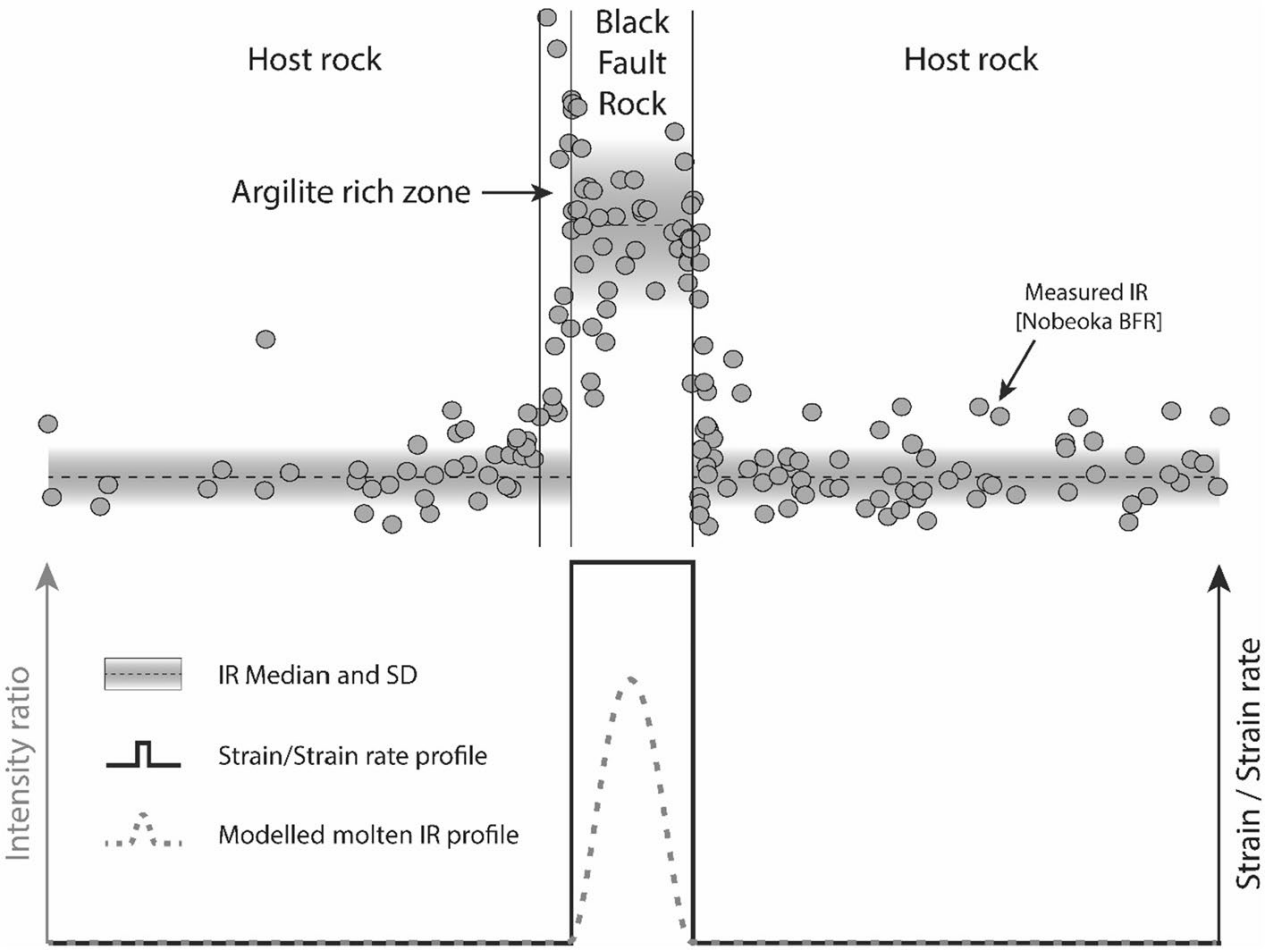
Intensity ratio profile and the amount of strain or strain rate across the host-rock and the Black Fault Rock for the Nobeoka case (with median and standard deviation shown as black dotted lines). Intensity ratio derived from thermal models of a viscously sheared molten layer (case (3)) is superimposed as a grey dotted line. A hypothetical strain/strain rate profile is shown as a black bold line. The stepwise shape of natural profile matches strain/strain rate profile, while it stands at variance with thermal models.

Parametric models presented here show that frictional melting and viscous shear produce Gaussian shape profiles where symmetric gradients in intensity ratio are located within the molten layer. By contrast, natural profiles follow rectangular function shapes with major discontinuities (i.e. steps) coinciding with Black Fault rocks/host-rock physical boundaries with up to 40% variation of the intensity ratio confined to few micrometers. Furthermore, towards the outer boundary of Black Fault Rocks, the modelled peak values are much lower than those in natural cases, even considering extreme conditions of slip and heat production (Figs. 6b and 7). Therefore, the sharp increase in Raman spectra properties of carbonaceous material in the Black Fault Rocks appears inconsistent and therefore not to be controlled by the high and short-lived temperature increase during seismic slip.

This conclusion is further supported in polystaged deformation faults such as the Black Fault Rock of Kodiak (Fig. 5 and Supplementary Fig. 2), where the presence of multiple steps in Raman Intensity ratio is difficult to reconcile with thermal models of multiple melting stages.

Other features of Raman Spectroscopy of Carbonaceous Material profiles are at variance with the hypothesis of a temperature control. First, intensity ratios retrieved from injection structures are much lower than those in the fault core, again in contradiction with a common origin as a quenched melt (Fig. 4). Second, the highest intensity ratio values are mostly measured on the rim of the fault core layer, which is colder than the fault core in thermal models because of heat diffusion. Third, the microstructures do not show evolution from the center of the vein to the rim, except a thinner grain size on the extreme rim, whereas the temperature profile shows a Gaussian shape evolution.

### Interpretation of microstructural observations in terms of formation process

The three Black Fault Rocks described in this study show features very close to the ones described for the melt-origin pseudotachylytes. These samples are composed of an ultrafinegrained matrix with micro-scale sub-rounded clasts of quartz and plagioclase. Some of these clasts show fractures and corroded shapes interpreted as resulting from interactions with a melt^11,12^. However, the same corroded shape of the plagioclase has been observed in cataclastic rocks from the Kodiak Accretionary Complex, while gulf of corrosion in quartz are described in the Nobeoka BFR host-rock (Supplementary Fig. 4). Moreover, the ultra-fine matrix material that composes the matrix of the Black Fault Rock cannot be considered as an undisputable evidence for the former presence of a melt. Indeed, microfault zones filled with ultra-fine matrix material have also been described in slow creep experiments where frictional temperature increase is not significant^16,17^. In addition, Ti–rich and Fe-rich particles, interpreted as droplets, have been described in the three samples^8,12–14^. Our observations confirm the presence of Ti–rich particles in the Black Fault Rock but their shape is systematically euhedral. We observed also in the Black Fault Rock the absence of Fe-rich particles, which are abundant in the host-rock, in the exception of the Nobeoka BFR where Fe-rich particles are still present. Incipient replacement of iron sulfides by Fe-rich oxides is already operative in the host-rocks, in the form of corona of Fe-oxides around iron-sulfide particles (Fig. 3d). The difference in the proportion of the Fe-rich and Ti–rich particles between host-rock and Black Fault Rock, induced by the destabilization of Fe-rich particles, can therefore be interpreted as a result of fluid-rock interactions under variable redox-conditions, associated with fluid circulation in fault zones^42^. Black Fault Rocks have also an increase porosity with respect to host-rock (Fig. 3). Increase in porosity might as well be the result of solid-state deformation, even at large depths, as demonstrated in viscously creeping crustal shear zones^43,44^. To finish, the presence of microlites, i.e. microcrystals crystallized from a melt, is not decribed in the three Black Fault Rocks considered here. The very fined grained phyllosilicates wrapping the clasts in the Black Fault Rocks have the same nature as the low-grade metamorphic mineral present in the host-rock^45^. In the Kodiak Black Fault Rock, micrometer-scale, euhedral and zoned plagioclase has been described^14^. The models to account for oscillatory zoning imply either coupling between mineral interface processes and elemental diffusion in the surrounding melt, or variations in temperature/melt composition related to convection in a magma chamber^46–49^. In both cases the formation of oscillatory zoning in plagioclase involves durations inconsistent with the temperature evolution of mm-thick molten layer (Fig. 6 and Supplementary Fig. 3). However, these oscillatory zones can be interpreted as an local evolution of temperature, pressure or water^50,51^ content and could be the result of intense fluid-rock reaction^21^.

As a conclusion, in the three Black Fault Rock described here, none of the microstructures can be considered as irrefutable evidence to discriminate between melting^5^ and strain-related ultra-comminution^6,16–18^ as a formation process.

### An alternative model to shear heating: Raman Spectroscopy of Carbonaceous Material (RSCM) ratio profiles record the distribution of strain

While microstructures are rather ambiguous as to the origin of the Black Fault Rocks, the RSCM signal, both in terms of values and spatial distribution, is not consistent with any scenario of frictional heating and melting^28–31^. Therefore, temperature is not the parameter that controlled the sharp spatial variations in the distribution of crystallinity of carbonaceous material observed in all the natural samples.

Alternately to temperature, strain might be the factor controlling RSCM signal variations. The RSCM intensity ratio used in this work has been shown in natural and experimental examples to be sensitive to deformation^30,32,52,53,35^.

The interpretation of the intensity ratio in terms of strain is nevertheless not straightforward for several reasons. (1) The parameters of deformation (total amount of strain, strain-rate), as well as the conditions of deformation (pressure, temperature, fluid abundance), are only partially known, especially in natural samples, but also in experimental samples where macroscopic variables might be prescribed but local variables are only inferred. (2) The evolution from disordered carbonaceous material to graphite is not a monotonic and simple process, but rather a combination of many elementary processes. This is reflected in the complex evolution of RSCM spectra with increasing metamorphic temperature^26,38,54^: in disordered carbonaceous material, from catagenesis to low-grade metamorphic conditions, increase in temperature results in the increase in Raman Spectroscopy of Carbonaceous Material intensity ratio. Subsequently, at higher-grade conditions, increase in temperature results in the decrease in intensity ratio, up to the graphite where defect bands, i.e., D bands are absent. Therefore, comparison should be preferentially be carried out on carbonaceous matter in the same range of crystallinity. Most existing studies deal with relatively well-ordered carbonaceous particles pertaining to the higher-grade metamorphic conditions^32,34,52,53,55^. In contrast, our work focuses on relatively disordered carbonaceous typically present in external domains of orogenic belts^38,45^.

In natural samples, relationships between strain and crystallinity of carbonaceous particles point rather to an increase in carbonaceous material ordering in viscous mylonites^52,53,55^. In these examples, the low strain-rate attested by the microstructures precludes any significant increase in temperature by shear heating. Beyond the total amount of strain, other parameters of deformation, such as strain-rate, are also likely to play a role: in high grades rocks from the Hidaka Belt, two opposite trends of evolution, i.e. towards higher and lower carbonaceous material ordering, were observed in two types of high strain zones^53^.

In addition, experiments can also shed some lights on the relationship between strain and carbonaceous material ordering. Low strain-rate experiments have correlated without ambiguity zones of high strain with higher carbonaceous material ordering^34^. High strain-rate deformation experiments, aimed at reproducing fault zone deformation, have also shown that zones of localized deformation have higher carbonaceous material ordering^28,30,32^, but without a clear quantitative connection between the parameters of slip and the RSCM signal evolution. The interpretation of these high velocity experiments in terms of strain- carbonaceous material ordering relationship is nevertheless difficult, as not only strain, but also temperature is increased in the domains where deformation localizes, so that the respective effect of strain and temperature are impossible to disentangle. In addition, the temperature increase estimation is difficult. In fact, for similar starting material, deformation apparatus and experimental conditions, the calculated temperature increases ranges from < 300 °C^32^ to more than 1000 °C^28^.

The processes behind this increase in crystallinity as a result of shear are active at the nano-structural scale. Shear is responsible for the coalescence of pores and the parallelization of the basic structural units that conducts to a more regular organization of the aromatic carbon layers and to widen carbon sheets^33,34^. Therefore, shear will directly catalyze the carbonization reactions and generate higher crystallinity.

In the studied samples, the shape of RSCM profile provides the strongest argument in favor of the effect of strain on carbonaceous material ordering. The stepwise RSCM intensity ratio profiles across the natural Black Fault Rocks coincides well with the microstructure profiles, without evolution from the rim to the center of the vein, which reflect strain or strain-rate distribution (Fig. 7). Conversely to temperature profiles, strain or strain-rate profiles can be discontinuous, as a result of dynamic weakening processes.

As a summary, from existing natural and experimental evidences, and from the characteristics of RSCM intensity ratio distribution, we conclude that carbonaceous material crystallinity therefore reflects at the first order mechanical processes instead of frictional temperature increase. The Black Fault Rocks are composed of extremely fine-grained material which may be in some cases down to amorphous state, as a result of comminution, i.e., a drastic mechanical work during fault slip. The increase of temperature during such deformation is not sufficient to influence the Raman Spectroscopy ratios within/outside the Black Fault Rocks, a feature that we use in the following to place upper bounds on the maximum temperature reached within the slipping zone.

### Upper bounds on temperature increase during slip based on the RSCM data and thermal and kinetics modelling

High velocity friction experiments have attempted to reproduce the frictional processes that occur during fault seismic slip^28,56,57^. A severe limitation of these experiments is that the normal and shear stress are a few MPas at most^57–61^, far from the deviatoric stress that prevails at depth. As a consequence, the power dissipated on experimental fault planes is much smaller than during actual seismic slip^62^ and large uncertainties remains as to the temperature reached in deep fault cores during earthquakes. First-order estimations have been made on the basis of albite and quartz microstructures^28,56^, however, as we reported above (Fig. 3) ambiguities remain as to the interpretation of such microstructures. In the following, we attempt to estimate the range of possible temperature increase based on the RSCM signal evolution and on kinetics modelling.

The main conclusion reached above, based on the shape of the IR ratio in the three BFR, is that such shape is the result of strain distribution, and that no thermal effect can be detected. In other words, if there had been any thermal imprint on IR signal, then it is undetectable, hence lower than the scattering of IR values within the BFR or the host rock, of the order of 0.06.

Considering a slip duration of 1 s (as Sibson^62^), and a 1 mm-thick slip zone where the temperature is fixed during slip, then the maximum temperature is 1075 °C, above which the thermal imprint becomes larger than IR scattering along the step-wise profiles (Supplementary Fig. 5).

High-velocity experiments at ambient pressure have shown that during flash heating of a polyphasic mixture, each phase behaves independently and according to its melting point as a pure phase^20,63^.Our estimate of upper bound on temperature, based on the RSCM, is higher than the melting point of albite, estimated from 900 to 1100 °C at 2 to 5kbar^51^ but is largely lower than the melting point of the quartz (*i.e.* 1730 °C)^64,65^.

As a consequence, the distribution of IR in the three fault zones examined here is incompatible with temperature as high as to completely melt the rock; melting, if it ever occurred, was only partial and limited to the feldspar and phyllosilicate fraction of the rock. Therefore, the corrosion structures observed on albite could be the results of the melt of this mineral, on the contrary to quartz microstructures which are the result of hydrothermal or dissolution–precipitation processes. Alternatively, considering the ambiguity of all observed microstructures, the three Black Fault Rocks considered here might also be the product of mechanical wear, without any significant temperature increase nor partial melting.

## Supplementary Information

Supplementary Information.
