# Supplementary material for: The impact of melt versus mechanical wear on the formation of pseudotachylyte veins in accretionary complexes
Source: Sci Rep. 2022 Jan 27;12:1529. doi: 10.1038/s41598-022-05379-5 (PMC8795392; doi:10.1038/s41598-022-05379-5)
Supplement: Supplementary file 1 — Supplementary Information. [file 41598_2022_5379_MOESM1_ESM.docx]

Supplementary information

**The impact of melt versus mechanical wear on the formation of pseudotachylyte veins in accretionary complexes**


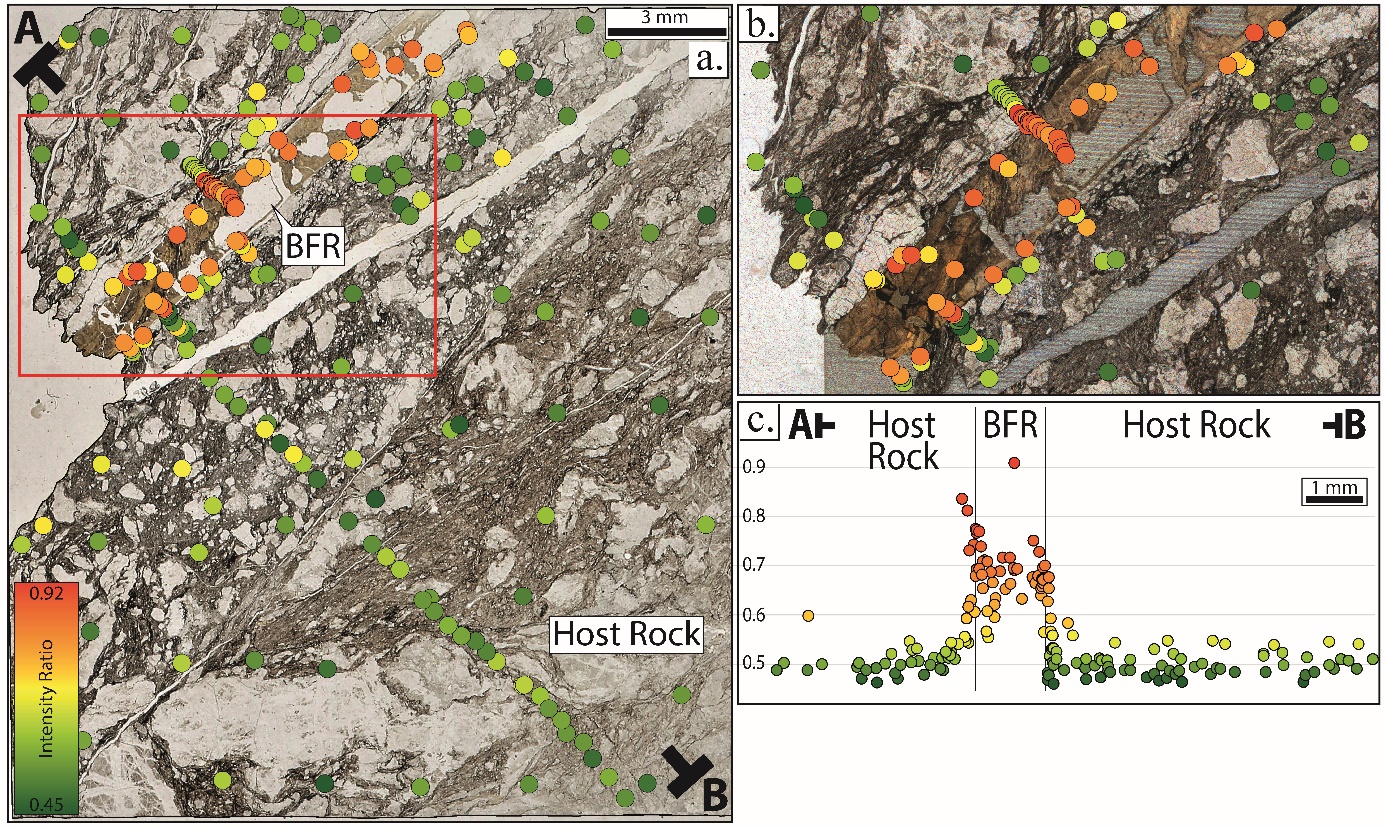


**Supplementary Figure 1 | Raman Spectroscopy results obtained for the Nobeoka Black Fault Rock** **a,** Distribution of intensity ratio over the sample – each dot corresponds to a carbonaceous particle measured by Raman spectroscopy **b,** Zoom in the contact of the Black Fault Rock with the host rock which shows discontinuity in the intensity ratio **c,** Cumulative cross-section showing the evolution of the intensity ratio perpendicular to the Black Fault Rock


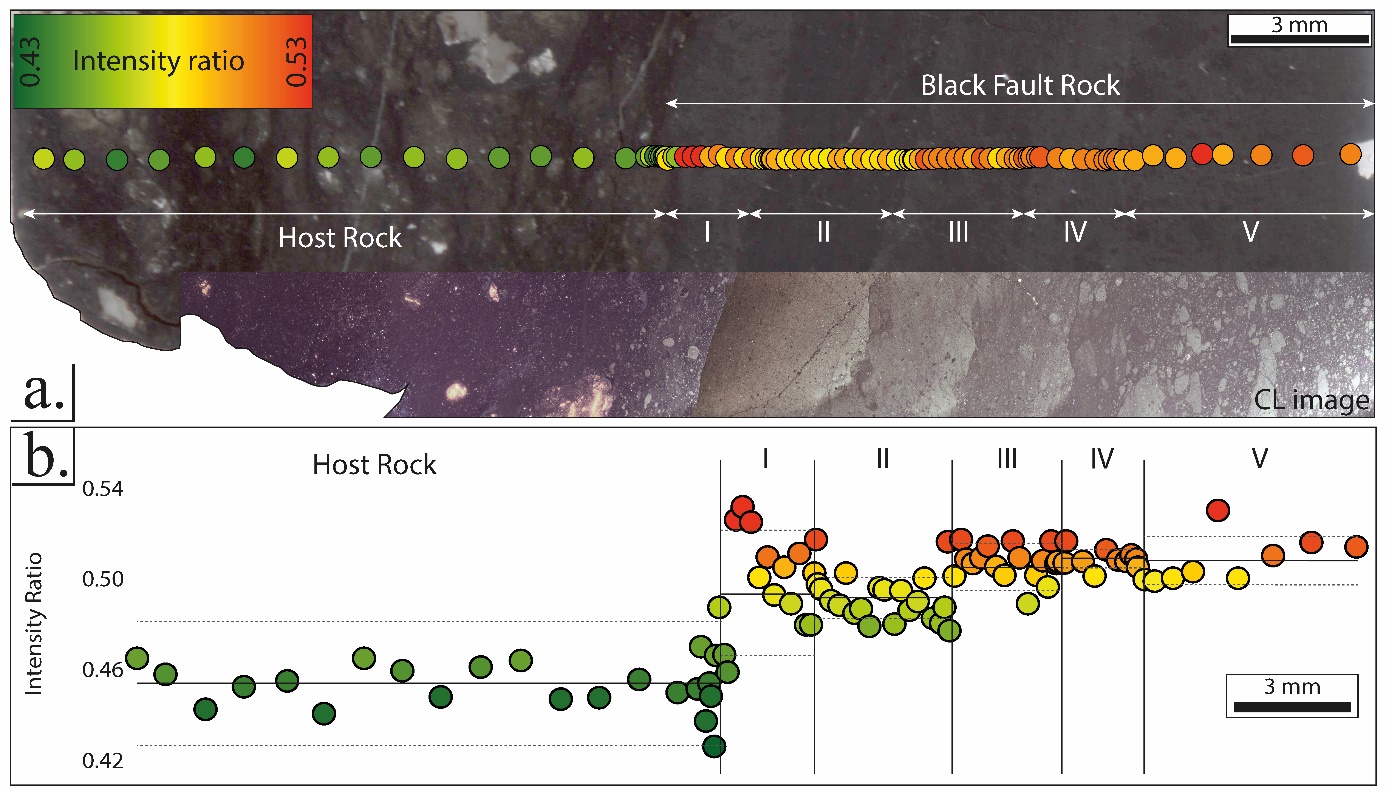


**Supplementary Figure 2 | Raman spectroscopy results obtained for the Kodiak Black Fault Rock described as a pseudotachylyte vein in Rowe et al., 2005 and Meneghini et al., 2010.** **a,** Distribution of the intensity ratio measured across the different microstructures and CL image that allows differentiating the multiple layers composing the Black Fault Rock **b,** Cross-section showing the evolution of the intensity ratio and area ratio through the sample. The largest discontinuities in intensity ratio coincide with the boundaries between the Black Fault Rock and host rock. Solid lines represent the mean intensity ratio and the dotted lines standard deviation of each unit.


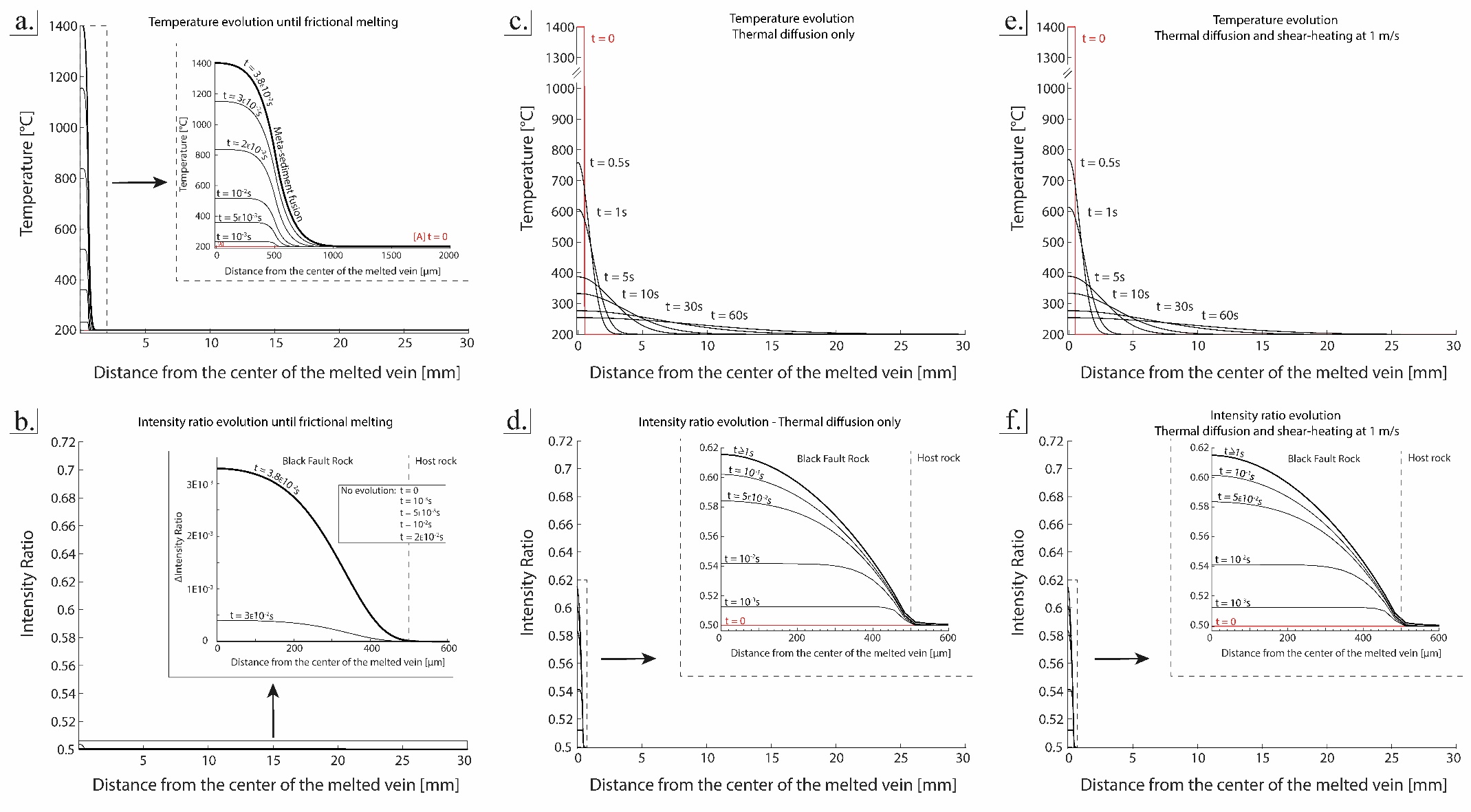


**Supplementary Figure 3 | Thermal and Kinetics modellings for different phases and scenarios during frictional melting . a, c, e,** Temperature and corresponding Raman Spectroscopy of Carbonaceous Material. **b, d, f**, Intensity ratio profiles across the molten layer and the host rock, for various times. **a, b,** Initial stage, with frictional melting until melting at 1400°C **c, d,** Following stage, with diffusional cooling of a 1400°C molten layer, without heat production **e, f,** same as c and d with heat production viscous shear. In **b, d, f,** for any time t and position x, the intensity ratio is calculated based on our kinetics model of organic matter maturation and the thermal history T(t).


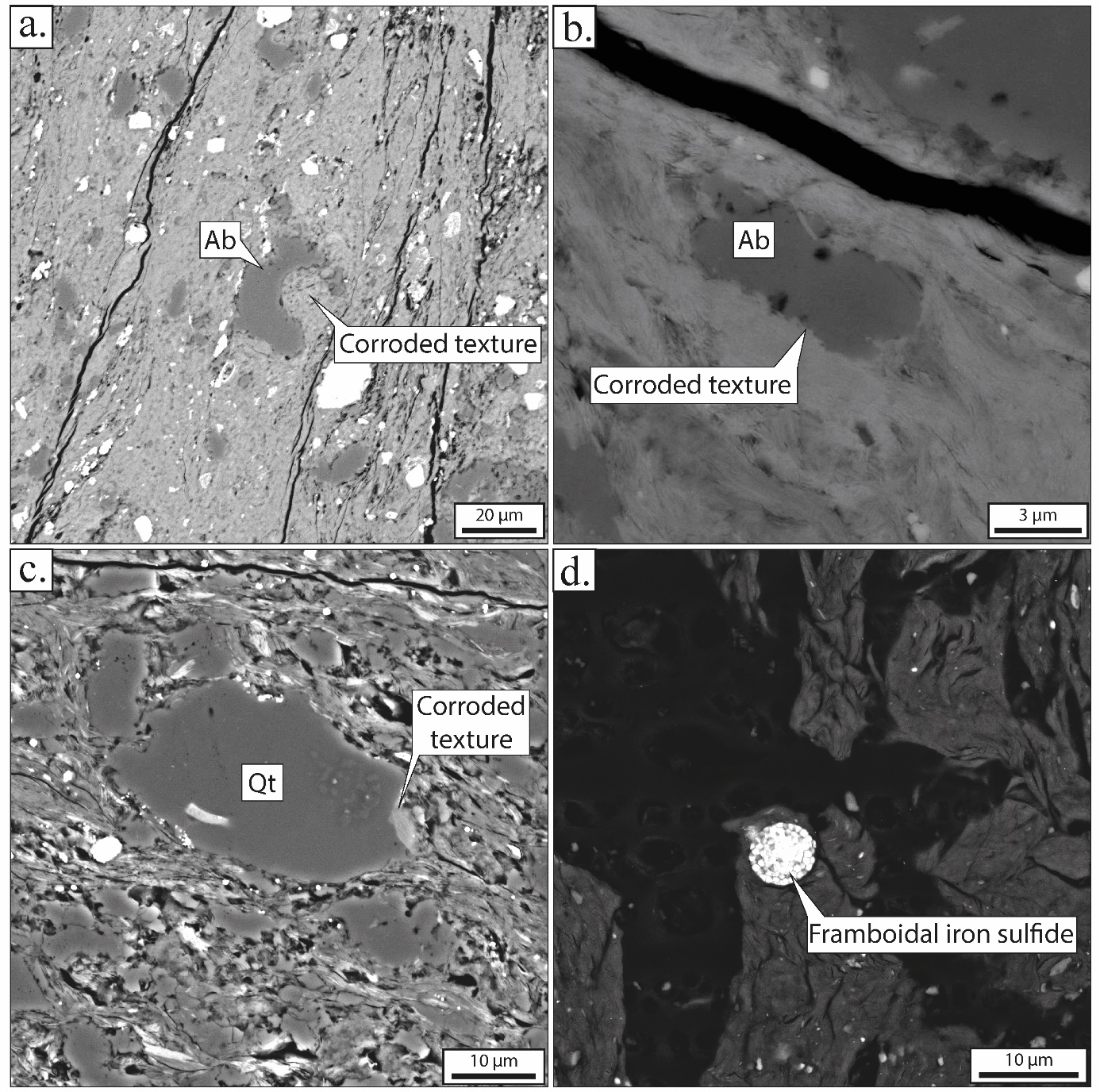


**Supplementary Figure 4 | Microstructures of BFR and host rocks** **a,** Gulf of corrosion on an albite grain observed in the cataclasite near the BFR, in the Pasagshak area. **b,** Eroded and corroded texture of an albite grain wrapped in a shaly matrix, in Ghost Rock Formation. **c,** Corroded shape of quartz grain in the Hyuga Tectonic mélange, host-rock of the Nobeoka BFR. **d,** Framboidal-shaped grain of iron sulfide in the Nobeoka BFR layer. **a, b,** Kodiak Accretionary Complex **c, d,** Shimanto Belt


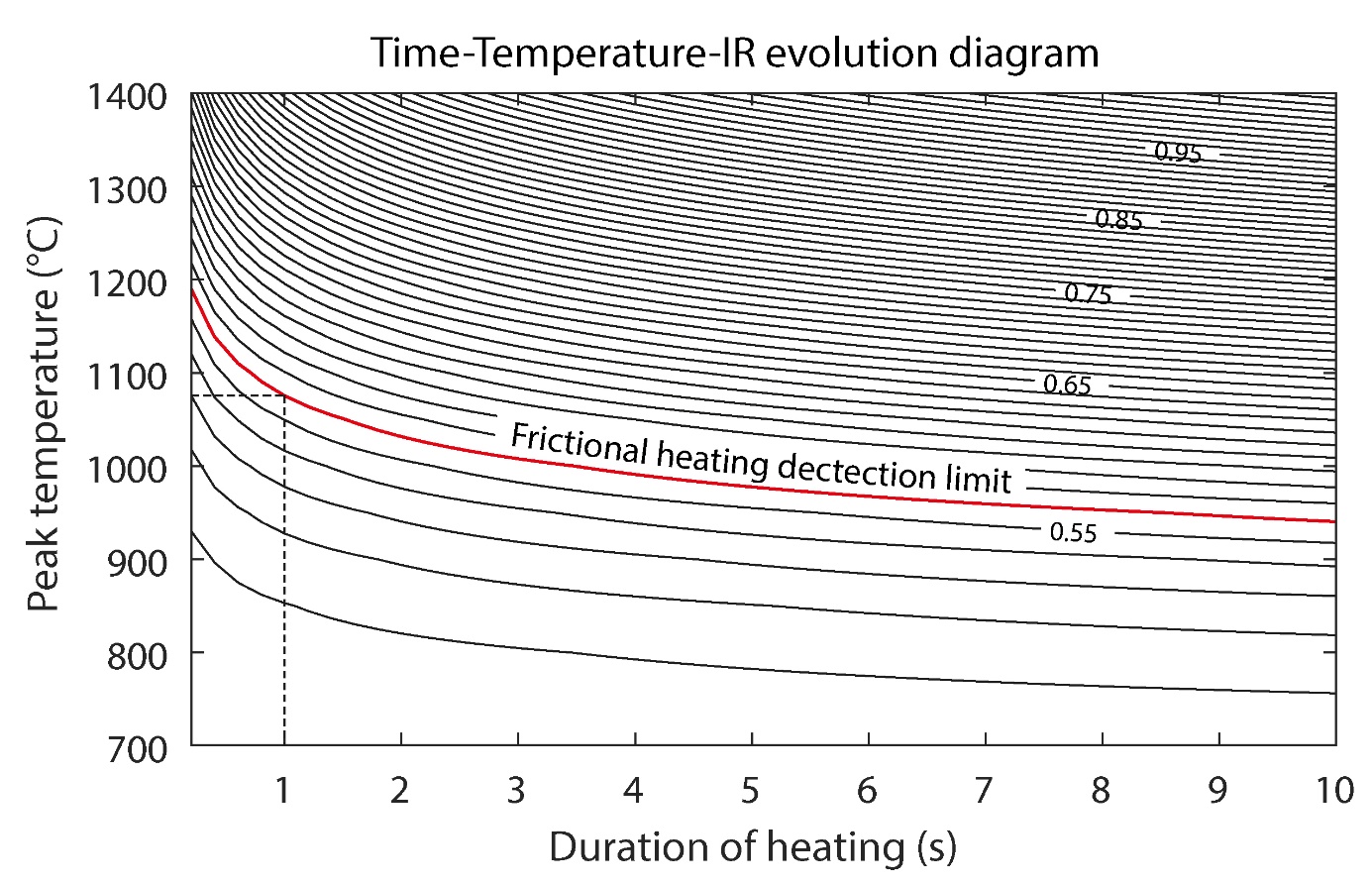


**Supplementary Figure 5 | T-t-IR diagram from the kinetics experiments on HN78.** Modelling of the IR evolution as a function of time and temperature showing the maximum temperature required to detect the frictional heating for a slip duration of 1 second.

**Supplementary Table 1 | Raman Spectroscopy parameters from maturation experiments on the sample HN78 from Hyuga coherent unit in Shimanto (Japan).** Estimated temp. = Apparent RSCM temperature obtained using Lahfid et al. (2010) and Beyssac et al. (2002) calibrations

**Supplementary Table 2 | Statistics on the intensity ratio obtained from the three Black Fault Rocks (BFR) analyzed in this study compared with their respective host-rock, used as a local reference (HR) and underformed references outside of the deformation zone.**

**Supplementary Table 3 | Location, structural and micro-structural observations of the three Black Fault Rocks analyzed in this study.**

|  |  |  |  |  | ***Textures*** | | | |  |  |
| --- | --- | --- | --- | --- | --- | --- | --- | --- | --- | --- |
| **Sample** | **Location** | **Latitude** | **Longitude** | **Geological unit** | **Fault core width** | **Single/Multi tectonic event** | **Fluidization structure** | **Ultrafine-grained matrix** |  |  |
| **Okitsu** | Japan | 33.219433 | 133.244452 | Okitsu mélange | 1 mm | Single | Yes | Yes |  |  |
| **Nobeoka** | Japan | 32.592666 | 131.735357 | Hyuga mélange | 1.2 - 1.5 mm | Single | No | Yes |  |  |
| **Kodiak** | USA | 57.422027 | -152.480361 | Ghost Rocks formation | 5 - 30cm | Multi | Yes | Yes |  |  |
|  |  |  |  |  |  |  |  |  |  |  |
|  | ***Micro- to nano-textures*** | | | | | | | | | |
| **Sample** | **Rounded clasts** | **Clasts grain size** | **Corrosion / Embayment structure** | **Flow texture** | **Matrix grain size** | **Fine-grains within matrix** | **Nature of fine-grains** | **Fine-grains size** | **Pores in the matrix** | **References** |
| **Okitsu** | Yes | < 100 µm | Yes | Yes | - | Yes | Rutiles | 100 nm - 3 µm | Yes | Ikesawa et al., 2003 |
| **Nobeoka** | Yes | < 0.1 mm | Yes | Yes | - | Yes | Fe-rich particles | 10 - 20 µm | No | Hasegawa et al., 2019 |
| **Kodiak ^[a]^** | Yes | 0.1 - 1 mm | Yes | Yes | < 2 µm | Yes | Tabular zoned plagioclases, Ti/Fe-rich particles | 1 - 2 µm | Rare | Rowe et al., 2005 Meneghini et al., 2010 |
|  | [a] The description corresponds to the homogeneous layer | | | | | | | | | |

Supplementary Note 1

**Assumptions of the thermal model**

The largest uncertainties in the thermal model lies in the heat production related to mechanical work, which constitutes a large fraction of the total faulting work^1^.

Heat production might in some cases be so large as to melt the slipping fault and form pseudotachylytes, as summarized in Di Toro et al. (2009)^2^. The result of melting (as well as other processes accompanying slip) is to lubricate the fault zone and to reduce sharply the shear stress on the fault plane^3,4^. After melting, heat production still persists and is the result of viscous shear-heating concomitant with slip.

Our model aims at assessing the effect of frictional melting on the carbonization of fault rocks. A quantitative modelling would require the precise knowledge of the heat production in space and time, incorporating the effect of frictional slip, melting (and associated shear stress decrease) and viscous shear. These processes and their coupling are not quantitatively constrained. Consequently, our model is based on a simplified scenario of melting, aiming at orders of magnitudes rather than accurate estimates of the variables at stake. We have decomposed the fault zone evolution into two stages: 1) The slip and heat production until the onset of melting in the fault core and 2) the slip and heat production after melting, when a layer of melt with a thickness equal to the thickness of the Black Fault Rock, is sheared between two solid blocks of rock. In this second stage, we consider independently the effects of heat diffusion from a molten layer and the viscous heat production, as the molten layer is being sheared.

- 1. Evolution of the system until melting

We consider here the effects of the frictional heat production that occurs until melting. The heat production is restricted to the layer that eventually melts (i.e. with the same thickness as the Black Fault Rock). The amount of volumetric energy generated by the frictional slip is estimated as:

$$\begin{aligned} \dot{E}= \tau.\dot{\varepsilon} \#\left( 1 \right) \end{aligned}$$

Where, $\tau$ is the shear and $\dot{\varepsilon}$ the slip rate divided by the size of the sheared layer. The shear stress is estimated from few MPa^5,6^ to a maximum of 10MPa^7,8^. Considering this value, for a 1 mm thick layer sheared with a slip rate at 10 m/s (see in the next section) the volumetric energy generated is 1.0e10^11^ J/m^3^/s.

Results have been presented in the Supplementary Figure 5a and 5b. Even considering a very large overshoot of the melting temperature of the phyllosilicates and feldspars^9,10^, melting is achieved very quickly. For example, a temperature as high as 1400°C is reached after 3.8.10^-2^ s. The critical distance, that consists in the distance of displacement before melting and slip weakening is about 30 cm and is totally comparable with the calculated critical distance for natural earthquake^11^. As a consequence of the very rapid temperature increase, the effect on carbonaceous material is not significant.

- 1. Evolution of the system after melting

2.1 Without viscous shear heating

After reaching the melting point thanks to the frictional melting, the next step is to consider the heat diffusion from a molten layer at 1400°C, an upper range of reported temperature in literature for experiments^2,12^. Such a high temperature is purposefully chosen as to enhance and highlight the possible effect of frictional heating on Raman signal. Supplementary Figure 5c and 5d presents the results obtained for the heat diffusion only: the temperature drops down very quickly and as a consequence there is a small evolution of the intensity ratio (0.615 in the core) inside the molten layer, still much lower than the values measured for the natural Black Fault Rocks (0.7 in median).

2.2 With viscous shear heating, considering displacement at 1m/s

Next step consisted to model the temperature field when a molten layer of the same thickness (h) as the Black Fault Rocks (i.e. 1mm in the model) is being sheared. We assume that the field of velocity in the molten layer is given by the Couette Flow equation^13^ and that the melt viscosity is uniform and constant in time.


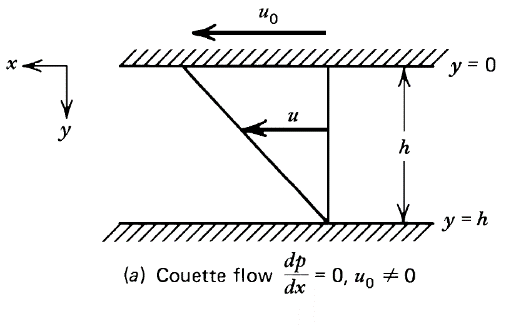


**Note figure 1 |** One-dimensional channel flows of a constant viscosity fluid (From Turcotte and Schubert, 1982)

The shear stress within the molten layer writes as:

$$\begin{aligned} \tau=\mu\frac{du_{x}}{dy} \#\left( 2.1 \right) \end{aligned}$$

Where µ is the viscosity and $u_{x}$ the velocity in the x direction. Based on the Couette Flow equation, for constant viscosity, the heat production (Eq. 3) yields:

$$\begin{aligned} \dot{E}= \tau.\dot{\varepsilon} = \tau\frac{d\left( u_{x} \right)}{dy}=\mu\left( \frac{u}{h} \right)^{2}\#\left( 2.2 \right) \end{aligned}$$

Viscosity of melt for rocks similar to the Black Fault Rocks range between 10^0^ and 10^2^ Pa.s^9^. The main factor that controls the amount of heat production is the slip velocity. Estimates for important earthquake^1,3,4^ (M>5) gave slip rate between 1 and 3 m/s. For example, during the mega-earthquake of Tohoku Oki, the maximum estimated slip rate was about 2.5 m/s^14^. There is a correlation between the slip rate and the size of the fault^15^. Fault zones where Black Fault Rocks are described in the accretionary prisms are structures internal to tectonic units, without major gap either in the stratigraphic ages or in paleotemperatures. For the Nobeoka Black Fault Rock, the sample comes from a second-order slip plane below the main slip plane of the Nobeoka Tectonic Line. As a consequence, these faults are second-order structures, with much lower slip rate than earthquakes on major, plate-boundary faults, such as 2011 Tohoku-Oki earthquakes.

In addition, melt is occurring from 0.1m/s based on natural example^16^ and from 0.4m/s on experimental examples^17^. According to these results, and in order to be consistent with studies focused on Black Fault Rocks in accretionary complexes, we consider here slip rates ranging from 0.1m/s to 1m/s.

Another way to obtain the average slip rate is to use the modelling function proposed by Madariaga and Olsen^18^.

$$\begin{aligned} Slip rate=u= \frac{\Delta\sigma}{G} \beta\#(3) \end{aligned}$$

The stress drop ($\Delta\sigma)$, the modulus of rigidity (G) and the shear wave speed (β) are estimated at 1 to 10MPa, 3.0.10^4^MPa and 3500 m/s^19^, respectively. Values obtained for the equation (3) are the same as previously found in the literature, as the slip rate calculated ranges between 0.2 and 1.2m/s. In order to consider the maximum heat production, we consider an upper bound on slip rate as 1m/s. The last parameter is the thickness of the molten layer, which is of the order of 1mm on the Black Fault Rocks studied. Based on these parameters we can estimate the maximum shear heating energy around 1.0e10^8^ J/m^3^/s.

The last parameter to specify is the duration of viscous shear-heating of the molten layer. Considering an upper bound on slip rate as 1m/s, as explained above, then shear heating duration can be estimated from the total displacement during the earthquake. For pseudotachylytes bearing faults, the total displacement of slip is estimated from 0.1 to 4 meters for earthquakes from M_5_ to M_8_^20^. According to Hasegawa^21^, and based on the thickness of the fault core similar to the one measured on the Gole Larghe fault in Italy, we can estimate the total displacement from 0.2 to 1.2 meters for a single tectonic event^22^. Some earthquakes can generate bigger slip displacement, like ~8 meters for the Chi-Chi earthquake^23^ or in extreme case, displacements as large as 50 meters for the M_9_ Tohuko Oki earthquake in 2011^14^. However, as discussed above, these earthquakes occurred on major tectonic faults, at the scale of tectonic plates, which do not compare to the much smaller faults hosting the Black Fault Rocks considered here. We consider therefore that ~ 5 meters for a single event is a more appropriate upper bound for the Black Fault Rocks^9,21,24^. Consequently, for a maximum fault displacement of 5 meters with a slip rate at 1m/s we obtain a slip duration of 5 second. We also consider that the viscous shear is effective during 5 second and drops to 0 after this duration. Supplementary Figure 5e and 5f reports the results obtained for the modelling considering the viscous shear heating with a slip rate at 1m/s. However, no significant difference can be observed in comparison with the model without viscous shear heating (2.1).

2.3 Budget of energy: cooling down vs. shear heating

The case study (2.1), where no viscous shear heating occurs, leads to variation in intensity ratio that are small with respect to measurements in natural Black Fault Rocks (Supplementary Figure 5c and 5d). Considering viscous shear heating for 5s at 1m/s (case 2.2) does not result in significant difference, because the production of heat is not sufficient. One can therefore wonder what conditions of slip might induce enough shear heating as to influence the intensity ratio of the carbonaceous matter.

In a first approach to this issue, one can simply consider the budget of heat associated with the cooling of the molten layer of 1mm of thickness, initially at T_melt_ and cooling down to the host-rock temperature T_host_:

$$\begin{aligned} E_{n}=Cp . \rho. h . Surf . \left( Tmelt-Thost \right)\#(4) \end{aligned}$$

For a square meter of surface, we obtain a total energy budget of 3.76e10^6^ Joules.

This budget can be compared with the amount of energy that can be produced by viscous shear heating. Based on the equation (2.2), the heat production scales with the square of slip velocity. An upper bound on heat production can be obtained using extreme slip velocity, of the order of 10 m/s^14^. Using equation (2.2), we can calculate the amount of energy produced by shear heating for slip duration in the range 0.1s to 1s and for slip rates in the range 1 m/s to 10 m/s, on a volume of V = h . Surf =1mm.1m^2^ (in Joules):

**Note table 1 |** Energy produced by shear heating in Joules for various slip duration and rate

|  | 1 m/s | 10 m/s |
| --- | --- | --- |
| 0.1 s | 1.0e10^4^ | 1.0e10^6^ |
| 1 s | 1.0e10^5^ | 1.0e10^7^ |

Comparing these values with the heat diffusion (E_n_), it appears that shear heating produces a significant amount of energy only for very high slip rates (10m/s) and for duration of at least 0.1s.

- 1. With viscous shear heating, considering displacement at 10m/s

On the basis of the calculations above, only high slip rate of 10m/s can possibly produce enough energy as to influence the intensity ratio of the carbonaceous material in the molten layer. In order to compare these modelling results with natural data we have to set the shear parameters to find the same value (eg. 0.7 for the intensity ratio) in the center of the molten vein. The appropriate slip rate and the slip duration are at 10 m/s and 1 second respectively. The total displacement for these parameters is 10 meters and is consistent with naturel estimated slip displacement during very large earthquakes. Results obtained have been presented in the Figure 3a and 3b of the main article.

Supplementary Note 2

**Material and methods**

1. Raman Spectra of Carbonaceous Material:

Samples were all cut in the X-Z structural plane to prepare 30 µm-thick standard thin sections. Sections were analyzed with ReniShaw InVIA microspectrometer using argon laser beam of 514 nm focused on the sample by DM2500 Leica microscope equipped with x100 objective. These apparatus are coupled with Renishaw software Wire 4.0 which was used for acquisition. Acquisition time was 30 to 200 seconds (Spectrometer opening time was around 5s with 40 spectra accumulation). Laser power was maintained at less than 0.5mW on the surface of the sample to avoid heating of carbonaceous material grain during analyses. To avoid defects bands generated during thin section polishing all analyzes were performed on subsurface grain by focusing laser beam through transparent or translucent minerals. Analyzes were processed along cross-section perpendicular to the fault core. Spacing between analyses were drastically reduced near the boundary of each Black Fault Rocks with host rock, from few tens of micrometers to less than 10 µm. Point coordinates were taken for georeferencing the analyses.

Raman spectra processing was done using PeakFit v4.12. In order to get the best comparison between carbonaceous material spectra from the host rock and Black Fault Rock only spectra with flat baseline were considered and linear baseline substraction function was applied. To determine the baseline, two bands of 100cm^-1^ of width were selected around 900 and 1900cm^-1^. To interpret Raman spectra and obtain Raman spectra parameters Lahfid et al. (2010) procedure was used for deconvolution. This approach is well adapted to low metamorphic grade and consist in a 5 bands (D1, D2, D3, D4 and G) deconvolution using Lorentzian functions. These bands form one Defect band (D band) centered around 1350cm-1 by compiling D4+D1+D3 and one Graphite band (G *sl.* Band) composed of D2 + G centered around 1600cm^-1^. By using area ratio RA1 = (D1+D4)/(D1+D2+D3+D4+G) it is possible to obtain an estimation of temperature in the range of 200 to 330°C with ±25°C uncertainty (RA1 = 0.0008 . T_max_ + 0.3758). In some cases, carbonaceous particles have higher crystallinity (i.e. Black Fault Rock spectra) so that the deconvolution using 5 bands described above was not possible. In such cases, we used the Beyssac et al. (2002) procedure and calibration using a 3 bands deconvolution. The main Raman spectra parameter used in this work was the intensity ratio (ie. R1 in Beyssac et al., 2002) which consists of comparing the height of the defect band and the raw graphite band (G *sl.* Band).

After the processing phase, using the GIS software ArcMap 10.6, each analysis was precisely located on microstructural pictures. Such high-resolution mapping allowed to compare micro-textures and the corresponding Raman spectra distribution.

1. Cathodoluminescence imaging (CL)

Cathodoluminescence (CL) has been used for the characterization of the microstructures for the mm-scale shear-zone in order to distinguish mineralogical and textural evolution along the strain gradient based on the luminescence of minerals mostly quartz, plagioclase and calcite because of the low/absent luminescence of clays and phyllosilicates of the matrix. CL was ran with a Cathodyne-Newtec apparatus using an automatized stage and coupled with a Leica microscope and a low light Qimaging Retica R1 camera. Thick section placed in a vacuum chamber at an Argon pressure ∼ 50 x 10^-3^ mbar and irradiated by a cold cathode source electron gun titled at 18° with the sample. Standard voltage-current beam conditions were maintained at 15kV and 150 µA during the acquisition time around 1 to 5 second per image.

1. Maturation in autoclave and kinetics modelling:

For the maturation experiments, a poorly mature CM shale from Hyuga coherent unit (HN78) estimated at 200°C using Raman Spectroscopy of Carbonaceous Materials has been used. We select this starting material based on similarities in terms of lithology and thermal conditions with the host-rock observed around the Black Fault Rock. All Raman Spectroscopy parameters (intensity ratio: IR (i.e R1 in Beyssac et al.) and area ratios: R2, RA1) the estimated temperature of this starting materials are available in the Extended Data Table 1. In addition, vitrain was used in order to follow maturation with a calibrated kinetic model of the Vitrinite reflectance^25^. Because the kinetics could be different between the type of carbonaceous material, we select a type III standard vitrain from the Candiota mine^26^. Before maturation, starting materials has been characterized using Raman spectroscopy, vitrinite reflectance and RockEval pyrolysis.

For the preparation of the experiments, shales and vitrain have been grinded into a fine powder .1g of shale powder and 120mg of vitrain separated by a gold leaf have beed loaded into Au-capsules of 50mm length and diameter Ø5.0-5.4. Au-capsules were welded close under Argon flow in order to get neutral atmosphere (<0.2% of O_2_) inside capsules and to avoid oxidation of carbonaceous material during maturation. Additionally, no water was added in the gold capsules for the same reason that means the only free water available is the one present in the starting material minerals.

Maturation experiments were carried out at respectively 500, 600, 700°C, and 1500 bar during 24 to 1080 hours. Two heating apparatus were used: one for experiments at 500 and 600°C with external heating system and H_2_O as a confining medium [A] and another one at 700°C with internal heating system and Argon as a confining medium [B].

At the end of each experiment, temperature was decreased very quickly in order to stop carbonization. Shale samples and vitrain were separated and prepared for Raman spectroscopy analysis. All the Raman parameters measured are available in the Extended Data Table 1.

Based on the Raman analyses of all experimental samples, we developed a kinetic model of carbonization to account for the evolution of the Raman intensity ratio parameter (IR) with temperature and time. We used the procedure of the superposition model described in Nakamura *et al.* (2017)^27^. The following sigmoid equation and variables were used:

$$\begin{aligned} f\left( T,t \right)= \frac{IRmin+\left( IRmax-IRmin \right)}{1+\left[ \frac{A\exp\left( \frac{-m}{T} \right)}{t} \right]^{h}} \#\left( 5 \right) \end{aligned}$$

**Note table 2 |** Constants obtained and used for the CM kinetics modellings

| Constant | Value |
| --- | --- |
| IR_min_ | 0.5 |
| IR_max_ | 1.62 |
| A | 1.04939 . 10^-8^ |
| -m | 27905 |
| h | 0.449 |

4. Numerical modelling:

4.0 Assumption of the model: laminar flow of the material

To simplify the problem to a single dimension (perpendicular to the fault layer) requires to assume that the flow of the material was laminar, so that all grains of carbonaceous material in the fault layer have preserved the same distance to the host rock throughout deformation, without redistribution by convection. The validity of this assumption can be assessed using the number of the Reynolds number of the flow, which can be defined as follows:

$$\begin{aligned} Re= \frac{\rho V Lc}{\mu}\#(6) \end{aligned}$$

**Note table 3 |** Constants used for the Reynolds number calculation

| Constant | Value | Unit | Reference |
| --- | --- | --- | --- |
| Density – ρ | 2660 | Kg/m^3^ | Schön, 1996^28^ |
| Slip rate – V | 1 - 10 | m/s | Cf Shear heating |
| Diameter – Lc | 1.10^-3^ | m | - |
| Viscosity – µ | 5 – 300 | Pa.s | Ujiie et al., 2007^9^ |

Thanks to these values (see Table above), this equation allows calculating a Reynolds number between 5.3 and 8.9e10^-3^. As the Reynolds number is lower than 2000, it means that the melt behavior was laminar, so that there were no mixing of the grains during the deformation and the problem can be appropriately described by a 1D approach. Such a laminar flow is further supported by the internal layering of the BFR, parallel to the planar boundaries of the BFR.

3.1 Thermal model

We describe the temperature field in a molten layer and its host rock using 1D numerical modelling of heat diffusion, including a source of heat production related to the work dissipated during rapid slip:

$$\begin{aligned} \frac{\partial T}{\partial t}= \kappa. \frac{\partial^{2}T}{\partial z^{2}}+\frac{S}{\rho. Cp} where \kappa= \frac{k}{\rho. Cp} \#\left( 7 \right) \end{aligned}$$

Where T is the temperature, t is the time, k is thermal conductivity, ρ is the density, Cp the heat capacity and S the heat production. All physical properties are considered as homogeneous throughout the profile. All the values used for this modelling are available on the table below.

Note that our thermal diffusion modelling without heat production was checked using the analytical solution provided in Carslaw and Jaeger (1959)^29^.

In this modelling, we always model the same time interval and spatial domain. It corresponds to a 10cm-thick domain of shale with an initial temperature of 200°C crossed by a 1mm-thick molten layer. The spatial resolutions is 1.10^-4^ meter along the modelling but we increase the resolution near the contact between the molten layer and the host rock at 3.10^-5^ meter. The temporal resolution has been set at 1.10^-4^ second.

**Note table 4** **|** Constants used for the heat diffusion modelling

| Constant | Value | Unit | References |
| --- | --- | --- | --- |
| Host rock size | 0.1 | m | - |
| Molten layer size | 1.10^-3^ | m | - |
| Host rock temperature | 200 | °C | - |
| Molten layer temperature | 1400 | °C | - |
| Thermal conductivity – k | 2.0700 | W/m/K | Cermak and Rybach, 1982^30^ |
| Heat capacity – Cp | 1180 | J/kg/K | Cermak and Rybach, 1982^30^ |
| Density – ρ | 2660 | Kg/m^3^ | Schön, 1996^28^ |

To finish, in this modelling, we consider that heat production is mainly generated by frictional and viscous shear-heating. However, others phenomena like the latent heat that occurs during the crystallization of the molten layer could control the temperature evolution by releasing heat. Based on the work of Bohrson and Spera^31^, we can estimate that the enthalpy production (H) ranges from 3.5 to 3.9e10^5^ J/kg^31^. For a 1mm thick layer on 1 square meter, the maximum heat diffusion corresponds 3.76e10^6^ Joules. Based on these data, we obtain an estimated enthalpy production of 9.31e10^5^ to 1.04e10^6^ Joules_._ Hence, the enthalpy production is four times lower than the heat possible diffusion (See the section 2.3 in Supplementary Note 1). Based on these results, we neglect the effect of latent heat release in the thermal modelling.

3.2 Carbonization of carbonaceous material modelling

In addition, the carbonization kinetics modelling established previously was integrated to the thermal modelling, in order to model the evolution with time of Raman spectroscopy parameter along the 1D profile.

Supplementary References

1. Scholz, C. *The Mechanics of Earthquakes and Faulting*. (Cambridge University Press, 1990).

2. Di Toro, G. & Pennacchioni, G. Pseudotachylytes and Earthquake Source Mechanics. in *Fault-Zone Properties and Earthquake Rupture Dynamics* 88–133 (Fukuyama, E., 2009).

3. Di Toro, G., Goldsby, D. L. & Tullis, T. E. Friction falls towards zero in quartz rock as slip velocity approaches seismic rates. *Nature* **427**, 436–439 (2004).

4. Di Toro, G. *et al.* Fault lubrication during earthquakes. *Nature* **471**, 494–498 (2011).

5. Lin, W. *et al.* Stress State in the Largest Displacement Area of the 2011 Tohoku-Oki Earthquake. *Science* **339**, 687–690 (2013).

6. Yoshida, K. *et al.* Stress before and after the 2011 great Tohoku-oki earthquake and induced earthquakes in inland areas of eastern Japan: STRESS CHANGE BY 2011 TOHOKU EARTHQUAKE. *Geophys. Res. Lett.* **39**, (2012).

7. Hardebeck, J. L. Coseismic and postseismic stress rotations due to great subduction zone earthquakes: STRESS ROTATIONS IN SUBDUCTION ZONES. *Geophys. Res. Lett.* **39**, (2012).

8. Hasegawa, A. *et al.* Change in stress field after the 2011 great Tohoku-Oki earthquake. *Earth Planet. Sci. Lett.* **355–356**, 231–243 (2012).

9. Ujiie, K., Yamaguchi, H., Sakaguchi, A. & Toh, S. Pseudotachylytes in an ancient accretionary complex and implications for melt lubrication during subduction zone earthquakes. *J. Struct. Geol.* **29**, 599–613 (2007).

10. Mukoyoshi, H., Sakaguchi, A., Otsuki, K., Hirono, T. & Soh, W. Co-seismic frictional melting along an out-of-sequence thrust in the Shimanto accretionary complex. Implications on the tsunamigenic potential of splay faults in modern subduction zones. *Earth Planet. Sci. Lett.* **245**, 330–343 (2006).

11. Ide, S. & Takeo, M. Determination of constitutive relations of fault slip based on seismic wave analysis. *J. Geophys. Res. Solid Earth* **102**, 27379–27391 (1997).

12. Spray, J. G. Pseudotachylyte controversy: Fact or friction? *Geology* **23**, 1119 (1995).

13. Turcotte, Donald. L. & Schubert, G. *Geodynamics*. (Cambridge, 1982).

14. Wei, S., Graves, R., Helmberger, D., Avouac, J.-P. & Jiang, J. Sources of shaking and flooding during the Tohoku-Oki earthquake: A mixture of rupture styles. *Earth Planet. Sci. Lett.* **333–334**, 91–100 (2012).

15. Kanamori, H. & Heaton, T. H. Microscopic and macroscopic physics of earthquakes. in *Geophysical Monograph Series* (eds. Rundle, J. B., Turcotte, D. L. & Klein, W.) vol. 120 147–163 (American Geophysical Union, 2000).

16. Sibson, R. H. Generation of Pseudotachylyte by Ancient Seismic Faulting. *Geophys. J. Int.* **43**, 775–794 (1975).

17. Tsutsumi, A. & Shimamoto, T. High-velocity frictional properties of gabbro. *Geophys. Res. Lett.* **24**, 699–702 (1997).

18. Madariaga, R. & Olsen, K. B. *Earthquake dynamics, International handbook of earthquake and engineering seismology*. vol. 81A (Elsevier, 2012).

19. Kanamori, H. & Brodsky, E. E. The physics of earthquakes. *Rep. Prog. Phys.* **67**, 1429–1496 (2004).

20. Sibson, R. H. Earthquake faulting as a structural process. *J. Struct. Geol.* **11**, 1–14 (1989).

21. Hasegawa, R. *et al.* Postseismic fluid discharge chemically recorded in altered pseudotachylyte discovered from an ancient megasplay fault: an example from the Nobeoka Thrust in the Shimanto accretionary complex, SW Japan. *Prog. Earth Planet. Sci.* **6**, (2019).

22. Di Toro, G. & Pennacchioni, G. Fault plane processes and mesoscopic structure of a strong-type seismogenic fault in tonalites (Adamello batholith, Southern Alps). *Tectonophysics* **402**, 55–80 (2005).

23. Ma, K.-F. *et al.* Slip zone and energetics of a large earthquake from the Taiwan Chelungpu-fault Drilling Project. *Nature* **444**, 473–476 (2006).

24. Ujiie, K. & Kimura, G. Earthquake faulting in subduction zones: insights from fault rocks in accretionary prisms. *Prog. Earth Planet. Sci.* **1**, 7 (2014).

25. Burnham, A. K. & Sweeney, J. J. A chemical kinetic model of vitrinite maturation and reflectance. *Geochim. Cosmochim. Acta* **53**, 2649–2657 (1989).

26. Silva, M. B. & Kalkreuth, W. Petrological and geochemical characterization of Candiota coal seams, Brazil — Implication for coal facies interpretations and coal rank. *Int. J. Coal Geol.* **64**, 217–238 (2005).

27. Nakamura, Y., Yoshino, T. & Satish-Kumar, M. An experimental kinetic study on the structural evolution of natural carbonaceous material to graphite. *Am. Mineral.* **102**, 135–148 (2017).

28. Schön, J. *Physical Properties of Rocks Fundamentals and Principles of Petrophysics*. vol. 65 (Elsevier B.V., 1996).

29. Carslaw, H. S. & Jaeger, J. C. *Conduction of Heat in Solids*. (Oxford University Press, 1959).

30. Cermak, V. & Rybach, L. *Thermal properties: Thermal conductivity and specific heat of minerals and rocks*. (G. Angeneister, 1982).

31. Bohrson, W. A. & Spera, F. J. Energy-constrained open-system magmatic processes IV: Geochemical, thermal and mass consequences of energy-constrained recharge, assimilation and fractional crystallization (EC-RAFC): OPEN-SYSTEM MAGMATIC PROCESSES. *Geochem. Geophys. Geosystems* **4**, (2003).
